# Protective Role of Sphingosine-1-Phosphate During Radiation-Induced Testicular Injury

**DOI:** 10.3390/antiox13111322

**Published:** 2024-10-30

**Authors:** Defan Wang, Renfeng Xu, Zhengchao Wang

**Affiliations:** 1 Fujian Provincial Key Laboratory of Reproductive Health Research, School of Medicine, Xiamen University, Xiamen 361102, China; defanwang@126.com; 2 Fujian Provincial Key Laboratory for Developmental Biology and Neurosciences, College of Life Sciences, Fujian Normal University, Fuzhou 350007, China; xurenfeng131772@126.com

**Keywords:** sphingosine 1-phosphate, reactive oxygen species, oxidative stress, inflammatory factor, testicular injury

## Abstract

The impact of ionizing radiation on the male reproductive system is gaining increasing attention, particularly when it comes to testicular damage, which may result in decreased sperm quality and hormonal imbalances. Finding effective protective measures to mitigate testicular damage caused by radiation has become a focal point in the biomedical field. S1P, an essential biological signaling molecule, has garnered significant interest due to its multiple roles in regulating cellular functions and its protective effects against radiation-induced testicular injury. S1P not only effectively reduces the generation of ROS induced by radiation but also alleviates oxidative stress by enhancing the activity of antioxidant enzymes. Furthermore, S1P inhibits radiation-induced cell apoptosis by regulating the expression of anti-apoptotic and pro-apoptotic proteins. Additionally, S1P alleviates radiation-induced inflammation by inhibiting the production of inflammatory factors, thereby further protecting testicular tissue. In summary, S1P effectively reduces radiation-induced testicular damage through multiple mechanisms, offering a promising therapeutic approach to safeguard male reproductive health. Future research should explore the specific mechanisms of action and clinical application potential of S1P, aiming to contribute significantly to the prevention and treatment of radiation damage.

## 1. Introduction

Radiation damage to the reproductive system, particularly the testicles, is a prevalent consequence of radiation therapy. Testicular injury not only impairs sperm production but also disrupts androgen secretion, thereby affecting male reproductive health and hormonal balance [1,2,3,4]. Recent studies indicate that ionizing radiation can significantly damage germ cells in the testes, resulting in diminished sperm quantity and quality [2,5,6]. These studies emphasize that radiation exposure not only causes DNA damage and oxidative stress responses but also induces cell apoptosis, thereby impacting spermatogenesis. Researchers have also found that certain antioxidants, such as alpha-lipoic acid and melatonin, can mitigate the extent of radiation-induced testicular damage [7,8,9,10,11,12]. The long-term effects of low-dose radiation on the testes are also areas of emerging concern. Animal studies indicate that even low-dose radiation may cause potential long-term damage to testicular tissue, affecting multiple stages of spermatogenesis [13]. Research has shown that chronic oxidative stress and inflammatory responses caused by low-dose radiation are primary contributors to abnormal sperm production [13,14]. Radiation not only affects germ cells but also exerts a substantial impact on testicular interstitial cells. Recent studies have shown that radiation can cause dysregulation of DNA repair mechanisms in testicular interstitial cells, thereby impairing the synthesis and secretion of testosterone [3,4]. Dysfunction of testicular interstitial cells could further exacerbate the negative effects of radiation on spermatogenesis [15,16].

S1P is a critical bioactive lipid that regulates cell survival, proliferation, and apoptosis [17,18,19,20]. Studies have demonstrated that S1P can activate multiple pathways, such as PI-3K/Akt and MAPK, by interacting with its specific receptors, thereby promoting cell survival [19,20,21,22]. These pathways enhance survival by inhibiting the expression of pro-apoptotic genes or activating anti-apoptotic genes. Research further indicates that S1P can promote cell cycle progression and increase cell proliferation by activating the Ras and ERK/MAPK signaling [23]. Additionally, S1P facilitates the G1 to S phase transition in cells by cell cycle proteins and CDKs [24]. Notably, the role of S1P in apoptosis is dualistic [25,26,27]. In some cases, S1P can suppress apoptosis by activating anti-apoptotic signaling pathways, such as NF-κB [25]. However, under certain conditions, S1P can also induce apoptosis by promoting the expression of pro-apoptotic factors or inhibiting survival signaling pathways [26,27]. The role of S1P is contingent upon the cell type, microenvironment, and the expression profile of S1P receptor subtypes. Research on S1P’s role in cancer is particularly active, with high levels of S1P being associated with poor prognosis in various cancers [28,29,30]. Therefore, the S1P signaling pathway has emerged as a promising target for anti-cancer therapies.

Our recent research not only demonstrates the role of S1P in maintaining testicular functions and steroid hormone balance [17] but has also revealed a protective effect of HIF-1α-mediated autophagy in radiation-induced testicular injury [6]. In addition, S1P can reduce the accumulation of ROS caused by radiation and protect testicular cells from oxidative stress via anti-apoptotic and antioxidant mechanisms [31]. Therefore, this review aims to summarize the protective effects of S1P on radiation-induced testicular injury and its potential mechanisms, which is significant for developing new preventive and therapeutic strategies to protect male reproductive health in clinical settings.

## 2. Radiation-Induced Testicular Injury

Radiation is the emission of energy as electromagnetic waves or subatomic particles, originating from natural sources like cosmic rays and radon, as well as artificial sources such as medical imaging and nuclear power, and can cause systemic damage by inducing oxidative stress, DNA damage, and cellular dysfunction, with particularly harmful effects on rapidly dividing tissues like those in the male reproductive system [32]. Research has shown that radiation can induce direct structural damage to testicular tissue, leading to apoptosis of spermatogenic epithelial cells and impairing spermatogenesis [14,33,34]. Asadi et al. reported the effects of X-ray radiation on testicular tissue, emphasizing its high susceptibility to X-ray exposure [35]. Fukunaga et al. analyzed the response of testicular tissue to varying radiation doses [36], while Qu et al. established a direct correlation between the degree of testicular injury and radiation dose [37]. These studies indicate that even low-dose radiation (1.4–2.6 Gy) can induce damage to testicular cells, potentially leading to permanent infertility [36,37,38]. Additionally, Ibrahim and Rakici et al. highlighted that therapeutic radiation can still exert deleterious effects on testicular tissue, underscoring the need for effective and safe radioprotective agents to mitigate gamma radiation-induced testicular damage, which remains a significant challenge in nuclear medicine [13,39].

**Figure 1:**
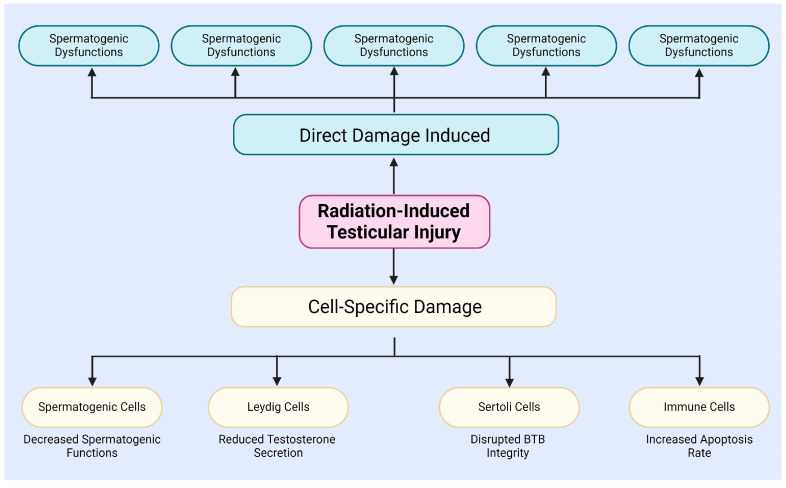
The testicular injury induced by radiation.

After radiation exposure, testicular tissue experiences extracellular matrix degradation in the interstitial compartment of the testis, BTB disruption, and germ cell DNA damage (Figure 1) [34]. The BTB is critical to the male reproductive system, safeguarding spermatogenesis from external insults. Formed by adjacent Sertoli cells, the BTB exhibits selective permeability, restricting the entry of harmful substances and xenobiotics [40,41,42]. Son et al. examined radiation-induced changes in BTB permeability and its role in male infertility [43]. Additionally, Zhang et al. demonstrated that NAD+, a crucial metabolite, can mitigate X-ray-induced DNA damage and structural changes in testicular tissue [44]. Amer et al. showed that melatonin can cross the BTB, modulate testicular immune responses, and alleviate radiation-induced inflammation [9].

Radiation exposure is known to induce substantial oxidative stress in testicular tissue, elevating the production of ROS, which leads to lipid peroxidation of cell membranes, protein damage, and DNA breakage [34,45,46]. Grewenig et al. investigated the molecular and cellular mechanisms underlying the pronounced radiation sensitivity in spermatogenesis, correlating radiation-induced DNA damage with the proliferation, differentiation, and apoptosis of testicular germ cells. They also found that radiation-induced DNA double-strand breaks posed the most significant risk to the genomic integrity of germ cells [47]. Cordelli et al. reported via DNA flow cytometry analysis a dose-dependent increase in testicular cell DNA damage 14 days post-radiation and a marked increase in sperm DNA strand breaks after 45 days [48]. Guo et al. demonstrated that radiation impairs the male reproductive system through mechanisms involving DNA damage, oxidative stress, and apoptosis, and highlighted the protective effects of various pharmacological agents against radiation-induced damage [49].

In addition, radiation induces unique effects in different types of cells within the testes, including spermatogenic cells, Leydig cells, Sertoli cells, and immune cells (Figure 1) [9,50,51,52,53,54,55,56]. For instance, radiation specifically damages spermatogenic cells, particularly spermatogonia and spermatocytes, mainly via the induction of DSBs and chromosomal aberrations, leading to a decline in spermatogenesis [50]. The study by Haines and Kesari et al. demonstrated a dose-dependent relationship between radiation dose and DNA damage in germ cells [57,58]. When the X-ray irradiation dose ranged from 0.25 to 4 Gy, DNA damage in germ cells significantly increased, with this damage being detectable in subsequent sperm [57,58]. Grewenig and Liu et al. found, through radiation experiments on mice, that even low doses of radiation can elicit both acute and chronic effects on spermatogenesis, resulting in a reduction in spermatogenic cell numbers and decreased sperm production [47,59]. Marjault and Li et al. observed that high-LET radiation not only increases oxidative stress levels in germ cells but also induces apoptosis and sperm DNA damage [45,60].

Radiation exposure not only activates the apoptotic pathway but also exacerbates damage to spermatogenic cells by modulating autophagy [51,52]. Interestingly, a study by Marjault and Yang et al. demonstrated that the unique spatial organization and hypoxic environment of spermatogenic cells in the seminiferous tubules provide a degree of protection against radiation [45,61]. However, spermatogenic cells remain highly sensitive to radiation, especially with respect to DNA damage [45,61]. 

Recent studies have shown that radiation exposure can lead to Leydig cell dysfunction, leading to decreased testosterone secretion and impaired support for the development of spermatogenic cells, ultimately reducing fertility [9,62]. Peak and Delic et al. found that the steroidogenic ability of Leydig cells decreased progressively with increasing radiation doses in cultured Leydig cells exposed to gamma radiation [63,64]. Research also indicates that the radiation threshold for Leydig cell dysfunction is approximately 5 Gy, with a gradual decrease in serum testosterone concentration within 2 weeks after exposure to doses exceeding this threshold. Following exposure to 15–20 Gy of radiation, testosterone levels became nearly undetectable, indicating that Leydig cells’ sensitivity to radiation is closely related to the dose [38,64]. De Felice and Georgakopoulos et al. observed that Leydig cells are significantly more sensitive to radiation in childhood compared to adulthood, with increasing tolerance observed with age [38,65]. Further research suggests that radiation not only directly affects steroidogenesis in Leydig cells but may also impair function by disrupting hormone signaling pathways [65,66].

Radiation can also impair Sertoli cells, thereby compromising BTB integrity, which in turn impairs the survival and differentiation of germ cells [50]. Research has shown that Sertoli cells protect post-meiotic germ cells from exogenous toxins by forming the BTB [37,67]; however, damage to Sertoli cells leads to collateral damage to germ cells, disrupting spermatogenesis [37,67]. Georgakopoulos et al. used data from animal experiments and clinical studies to demonstrate the extent of damage to Sertoli and germ cells caused by different radiation doses, along with the radiation-induced endocrine abnormalities [65]. Amer et al.’s study found that Sertoli cells were damaged by radiation, resulting in impaired spermatogenesis. Although Leydig cells exhibit considerable resistance to radiation, damage to Sertoli cells significantly affects the entire process of spermatogenesis [9].

Moreover, recent research indicates that radiation exerts notable cytotoxic effects on SSCs in the testes, leading to a marked reduction in stem cell numbers and impaired differentiation capacity [53,54]. The study by De Felice et al. reported that germ cells can self-renew to maintain population stability, and radiation-induced damage to these stem cells may have enduring consequences for sperm production capacity [38]. Rakici et al.’s research highlighted the profound effects of radiation on germ cells, revealing that even low-dose radiation can lead to reproductive health issues [13].

Additionally, immune cells in the testes, such as macrophages and dendritic cells, experience notable functional alterations post-radiation exposure, contributing to increased apoptosis of spermatogenic cells [55,56]. Georgakopoulos et al. highlighted that radiation induces alterations in the immune system, thereby affecting the immune privilege and reproductive capacity of the testes [65]. Hedger’s research demonstrated that Sertoli cells play a crucial role in maintaining testicular immune privilege [12]. Radiation has the potential to disrupt the immunosuppressive properties of these cells, leading to an enhanced immune response and adversely affecting reproductive function [68]. Gong et al. observed in testicular biopsies of infertile patients that the predominant infiltrating immune cells were CD3+T cells, while CD20+B cells were fewer in number, suggesting that radiation may promote abnormal T cell infiltration, leading to inflammation and tissue damage [69]. Bhushan and Zhao et al. examined the phenotypic alterations of various immune cell subtypes in the testes and the differences in their functions under both steady-state and inflammatory conditions. The findings indicated that radiation can modulate immune responses by altering specific immune cell interactions [70,71]. Said et al.’s study showed that immune cells in the testes exhibit functional changes following radiation exposure, exacerbating inflammatory reactions and negatively impacting spermatogenesis [7].

In summary, radiation not only induces direct damage to testicular tissue and cells through mechanisms such as DNA damage, apoptosis, autophagy, and oxidative stress [32,33,34], but also affects different types of testicular cells through cell-specific mechanisms, thereby impairing spermatogenesis and hormone secretion (Figure 1) [51,52,53,54,55,56]. These observations indicate that radiation-induced testicular damage is a complex, multi-level process, which will contribute to a deeper understanding of radiation-induced reproductive disorders and their potential regulatory mechanisms.

## 3. Sphingosine 1-Phosphate Signaling Pathway

**Figure 2:**
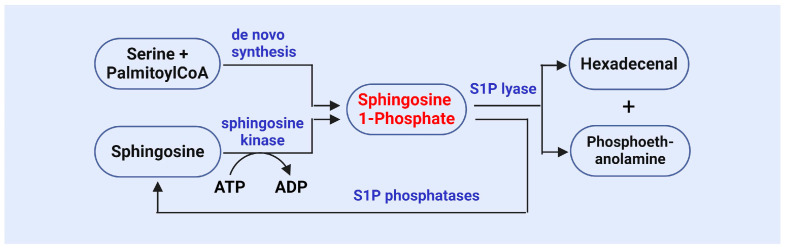
The synthesis and metabolism of sphingosine 1-phosphate.

S1P is an important bioactive lipid molecule catalyzed by SphK (Figure 2) [72,73,74,75]. Research has shown that SphK1 and SphK2 are key enzymes involved in the generation of S1P, as they phosphorylate sphingosine to produce S1P [72,73,74,75]. SphK1 primarily functions in the cytoplasm, while SphK2 is primarily localized in the nucleus. The functional regulation of these two kinases has a significant impact on cell survival, proliferation, and apoptosis [72,73,74,75]. A study on SphK revealed its role in the stress response, particularly under conditions where cells are subjected to oxidative stress or other external pressures. The activity of SphK1 is enhanced, leading to an increase in S1P levels. This stress-induced S1P generation is considered one of the protective mechanisms for cell survival [76].

Recent studies suggest that SphK expression and activity are regulated by various intracellular/extracellular signals, including growth factors, cytokines, and environmental stress [77,78]. Lebman et al. demonstrated that TGF-β upregulates SphK enzymatic activity and may also affect its expression by directly modulating SphK activity [79]. Bu et al. found that growth factors, such as EGF, regulate SphK1 expression and activity through signaling pathway activation, impacting inflammatory responses and immune cell function [80]. Gellings Lowe et al. showed that NGF induces an increase in SphK activity, suggesting its critical role in nerve cell survival and development [81]. Spiegel and Milstien reported that SphK1 modulates the expression of pro-inflammatory cytokines and nitric oxide in activated microglia [82]. Chen et al. demonstrated that SphK1 expression is linked to estrogen response, and its upregulation in inflammatory microenvironments has the potential to promote tumor progression [83]. Alemany et al. showed that cytokines modulate SphK activity by regulating Ca^2+^ release, thereby influencing the inflammatory response and cellular physiological function [84]. Nayak et al. found that SphK1 regulates pro-inflammatory cytokine expression through NF-κB pathway during acute inflammation, with N, *N*-dimethylsphingosine (DMS) effectively reducing SphK1 expression in mouse models [85]. Kim and Sieburth demonstrated that SphK activity is significantly enhanced in response to stress, such as nutrient deficiency, promoting autophagy and ensuring cell survival [86].

The metabolism of S1P is stringently regulated, and its metabolic balance is maintained by S1P phosphatase and S1P lyase (Figure 2), which reduce S1P levels and ensure homeostasis both intracellularly and extracellularly [19,20,21,87]. Research has demonstrated that S1P phosphatase can dephosphorylate S1P, resulting in sphingosine production, thereby regulating the concentration of S1P in cells and influencing its signaling pathways [19,20,21,87]. Abnormal activity of S1P phosphatase has been implicated in several diseases, including metabolic syndrome, cancer, and autoimmune disorders [20,88]. SPL irreversibly degrades S1P into ethanolamine phosphate and trans-2-hexadecenol, serving as the final step in S1P metabolism and playing a crucial role in maintaining S1P balance [19,20,21,87,88,89]. The activity of SPL in cells is regarded as the primary “outlet” for S1P, and its regulation can reflect cellular responses to environmental changes [90,91]. Olivera et al. found that deletion of the Sgpl1 gene in a murine model leads to significant S1P accumulation, highlighting the critical role of SPL in maintaining S1P metabolic balance [92]. Additionally, SPL participates in the degradation of S1P and plays pivotal roles in multiple physiological processes, including vascular regulation [93], immune responses [94], and tumorigenesis [95].

Recent studies have demonstrated that the metabolism and degradation of sphingosine-1-phosphate (S1P) are modulated by various signaling molecules, including cytokines, oxidative stress, and inflammatory factors [18]. Lebman et al.’s study suggests that the interactions between S1P and various cytokines are crucial in regulating cellular physiological and pathological processes [79]. Maceyka et al. analyzed the role of sphingolipid metabolites, particularly ceramides and S1P, as key signaling molecules in numerous cellular processes, emphasizing their importance in cytokine signaling transduction [96]. Sun et al. found that S1P can affect the chemotactic stimulation of lymph nodes by inhibiting the entry of chemokines into lymphocytes, highlighting its significant role in immune regulation [97]. Oxidative stress not only affects the generation of S1P but can also modulate its metabolism by altering the expression and activity of metabolic enzymes. This bidirectional regulatory mechanism may have a significant influence on various pathological states [98]. In non-alcoholic fatty liver disease (NAFLD), inhibition of S1P production exacerbates damage induced by oxidative stress [99]. The importance of S1P and its receptors in regulating inflammation has been widely documented. S1P not only promotes the expression of pro-inflammatory molecules but also regulates the synthesis of anti-inflammatory molecules, suggesting that it may serve a dual function in inflammatory responses [100,101]. The S1P receptor family is integral to the regulation of diverse immune cell functions, and S1P plays a key role in inflammatory responses by binding to these receptors, affecting the migration and activation of immune cells [18,102]. These signals can modulate the metabolic balance of S1P by adjusting the activity of S1P metabolism-related enzymes, thereby affecting cellular survival, proliferation, and apoptosis [18,103].

Research has identified that abnormalities in the S1P metabolic pathway are closely associated with various diseases, suggesting that disruption of S1P metabolic balance may contribute to the onset or progression of these conditions [21]. Grassi et al. demonstrated that S1P metabolism is tightly regulated, with sphingosine kinases (SK1 and SK2) playing distinct roles in different diseases, thereby highlighting the significance of these enzymes in metabolic disorders and related pathologies [101]. Duan et al. noted that alterations in the S1P axis, encompassing its generation, transport, and receptor interactions, can influence metabolism and mediate the development of metabolic diseases, such as obesity and diabetes [104]. Furthermore, Chen et al. revealed that S1P functions as a sphingolipid with both paracrine and autocrine effects, linking it to obesity, insulin resistance, hyperglycemia, and dyslipidemia [105]. Consequently, the development of pharmacological agents targeting S1P metabolism-related enzymes has emerged as a critical area of investigation.

**Figure 3:**
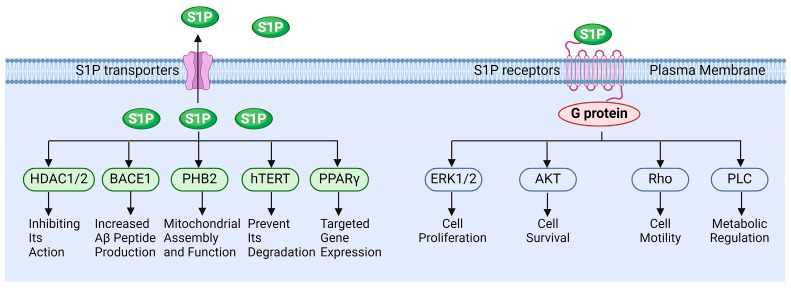
The functions of sphingosine 1-phosphate signaling.

S1P functions in various pathophysiological processes. Extracellular S1P is primarily exerted through binding to specific receptors, while intracellular S1P can also regulate cellular functions by directly interacting with target proteins (Figure 3). Wang et al. demonstrated that S1P regulates the molecular mechanisms underlying cancer cell survival, proliferation, and anti-apoptosis by activating downstream signaling of ERK, PI-3K/Akt, and NF-κB, etc., via its receptors [106]. Fan et al. reported that S1P activates the Akt/PKB and ERK signaling pathways through binding to the S1PR1 receptor, thereby modulating endothelial cell survival and angiogenesis. This suggests that the S1P-S1PR1 signaling pathway is essential for endothelial cell anti-apoptosis and the promotion of angiogenesis [107]. Alaamery and Wieczorek et al. elucidated the mechanism by which S1P and its receptor regulate apoptosis in neurodegenerative diseases [108,109]. Jozefczuk and Ouyang et al. systematically analyzed the role of S1P signaling in cardiovascular disease and elucidated its potential as a therapeutic target [110,111]. However, the role of S1P signaling in the reproductive system remains insufficiently elucidated, particularly concerning the regulation of testicular functions by S1P.

## 4. Protective Role of S1P Against Radiation-Induced Testicular Injury

**Figure 4:**
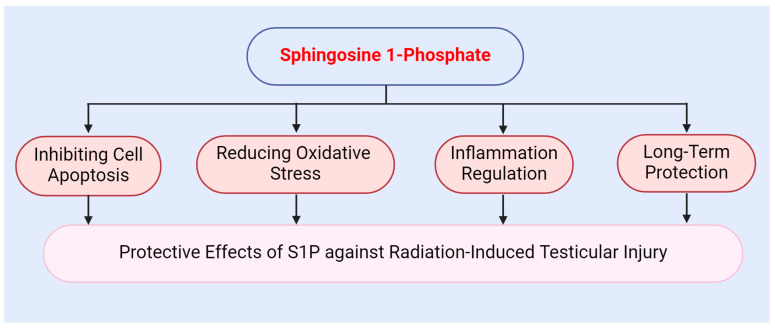
Protective role of sphingosine 1-phosphate against radiation-induced testicular injury.

The protective mechanisms of S1P against radiation-induced testicular injury operate at multiple levels. Firstly, S1P exhibits a potent anti-apoptotic effect. Secondly, S1P modulates intracellular antioxidant responses via its receptors. Additionally, S1P alleviates the inflammatory response induced by radiation. Overall, S1P provides effective protection for the testes and mitigates radiation-induced damage through various mechanisms, including antioxidant, anti-apoptotic, and anti-inflammatory effects (Figure 4).

### 4.1. The Role of S1P in Inhibiting Cell Apoptosis

S1P activates multiple downstream signaling pathways, including PI-3K/Akt, MAPK, and NF-κB, through its receptors, thereby modulating intracellular apoptotic signaling pathways and inhibiting cell apoptosis [19,20]. Research conducted by Castillo et al. indicates that ERK activation is essential to S1P’s anti-apoptotic effects [112]. In contrast, Maceyka et al. demonstrated that sphingosine and its metabolite S1P exert opposing effects on cell proliferation and apoptosis, with ceramide typically promoting apoptosis while S1P stimulates growth and inhibits it [27]. Furthermore, Giussani et al. found that S1P exerts its anti-apoptotic effect by activating ERK and inhibiting ceramide-mediated apoptosis [113]. Zou et al. reported that S1P inhibits neutrophil apoptosis through Gi/o and downstream p38 MAPK signaling [114]. Additionally, MAPK signaling inhibitors, such as U0126, SB203580, and SP600125, have elucidated the role of the MAPK pathway in S1P-mediated anti-apoptotic signaling [115].

S1P promotes cell survival signaling by modulating cell membrane fluidity and intercellular interactions [27,28]. For instance, Nix and Stoffel investigated the role of lipids in cell membrane fluidity, with particular focus on sphingosine and S1P. Their results demonstrated that sphingosine decreases T cell proliferation and renders these cells more susceptible to apoptosis, whereas S1P enhances cell survival and proliferation by increasing cell membrane fluidity [116]. Maceyka et al. also highlighted that sphingosine and its metabolite S1P significantly influence cell membrane fluidity. The generation of S1P facilitates cell proliferation and inhibits apoptosis, while sphingosine exerts the opposite effect, underscoring the importance of both molecules in cellular decision-making regarding life and death [27]. Furthermore, additional studies have indicated that S1P can modify the composition and fluidity of cell membranes through its receptors, thereby altering the cellular microenvironment and playing a protective role in response to various cellular stressors [29,117,118,119].

Additionally, S1P regulates intercellular signaling by binding to cell surface receptors, thereby promoting cell survival and inhibiting apoptosis. This underscores the significance of S1P in facilitating intercellular interactions [120]. Rutherford et al. found S1P receptor 1 expression in CCL39 lung fibroblasts enhances resistance to apoptosis following the withdrawal of growth factors, highlighting the critical role of S1P receptors in mediating intercellular interactions and survival signaling [22].

Recent studies have demonstrated that S1P promotes the expression of anti-apoptotic proteins, such as Bcl-2, by activating the PI-3K/Akt signaling pathway, while simultaneously inhibiting the activities of pro-apoptotic proteins, including Bax and Caspase-3 [26,121]. This mechanism effectively prevents both the initiation and progression of apoptosis. Furthermore, Li et al. investigated the protective effect of S1P on granulosa cell apoptosis, revealing that S1P inhibits chemotherapeutic agent-induced apoptosis by activating the PI-3K/Akt pathway [122]. The application of LY294002 completely abolished the protective effect of S1P, thereby confirming the critical role of PI-3K/Akt signaling in this context [122].

Furthermore, sphingosine-1-phosphate (S1P) enhances cellular resistance to apoptotic stimuli and inhibits inflammatory factors through the activation of NF-κB [18]. This activation is crucial for preventing apoptosis and preserving tissue function. For instance, S1P can enhance NF-κB signaling activity by binding to TRAF2, thereby promoting cell survival [123,124]. Additionally, several studies have demonstrated that S1P reduces inflammation and oxidative stress by inhibiting the SphK1/S1P/NF-κB signaling pathway, which in turn mitigates cell apoptosis [97].

These findings illustrate the complex interplay between the NF-κB and S1P signaling pathways [125]. S1P not only inhibits cell apoptosis but also plays a significant role in regulating macrophage phenotypes and mediating inflammatory signals [126].

### 4.2. The Role of S1P in Reducing Oxidative Stress

**Figure 5:**
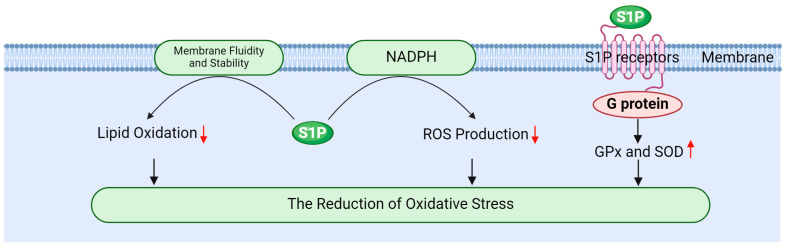
The role of sphingosine 1-phosphate in reducing oxidative stress.

S1P effectively mitigates oxidative stress through multiple mechanisms, thereby safeguarding testicular cells from radiation-induced damage. Its primary mechanisms include enhancing antioxidant enzyme activity, inhibiting ROS generation, and stabilizing cellular membranes (Figure 5). 

Firstly, S1P activates PI-3K/Akt signaling pathway via its receptors, promoting the expression and activity of antioxidant enzymes such as GPx and SOD [20,127]. Gurgul Convey’s research indicates that S1P plays a protective role in pancreatic beta cells against oxidative damage by regulating the expression of these antioxidant enzymes [98]. Furthermore, Sun et al. demonstrated that S1P indirectly influences antioxidant enzyme activity by enhancing glycolysis and oxygen transport in red blood cells. This metabolic shift can lead to increased activity of antioxidant enzymes, thereby improving the overall antioxidant capacity of cells [128]. Elevating the activity of these enzymes is crucial for neutralizing ROS, reducing oxidative stress, and effectively protecting testicular cells from radiation-induced damage [17,19,78].

Secondly, S1P mitigates ROS production by modulating the intracellular redox state. It inhibits ROS-generating enzymes, such as NADPH oxidase, through the regulation of signaling pathways at the cell membrane, thereby reducing oxidative stress [16,87,129]. Testicular tissue relies on its endogenous antioxidant enzyme system to counter radiation-induced oxidative stress, with SOD and GPx serving as the primary antioxidant defense enzymes responsible for scavenging ROS and protecting cells [35,130]. SOD plays a key role in the cellular response to oxidative stress by catalyzing the dismutation of O^2−^ into H_2_O_2_ and molecular oxygen, a process that is particularly crucial in testicular tissue [130,131]. GPx, in turn, catalyzes the reduction of hydrogen peroxide and organic peroxides, shielding cells from oxidative damage. This enzyme is integral to spermatogenesis and the maintenance of normal testicular function [131,132].

Finally, S1P alleviates the oxidation of membrane lipids by modulating the fluidity and stability of cellular membranes. Membrane stability is vital for preserving cellular function and resisting oxidative damage; thus, S1P plays a significant role in mitigating oxidative stress-related damage to cells [9,13,16]. For instance, ionizing radiation can induce the production of ROS, which can lead to lipid peroxidation, DNA damage, and subsequent apoptosis. These detrimental processes can impair spermatogenic function and hormone secretion in the testes, resulting in testicular atrophy and reduced fertility [5,39]. However, S1P influences cell function and survival by regulating both ROS production and clearance [133].

### 4.3. The Role of S1P in Inflammation Regulation

**Figure 6:**
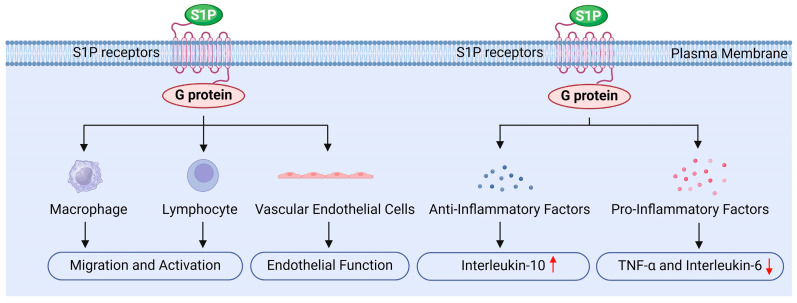
The role of sphingosine 1-phosphate in inflammation regulation.

S1P may also regulate inflammation, primarily by influencing various intracellular signaling pathways through its receptors, thereby modulating immune cell function and inflammatory signaling (Figure 6) [8,18,19,34]. 

Firstly, S1P influences the migration and activation of immune cells by binding to its specific receptors. It promotes the migration of white blood cells, macrophages, and lymphocytes, while regulating their accumulation and function at sites of inflammation, which is crucial for initiating and sustaining the inflammatory response [18,19]. The studies by Bravo and Chatzikonstantinou et al. demonstrated that S1P binding to its receptors can regulate the differentiation, activation, and survival of immune cells, particularly within the adaptive and innate immune systems [134,135]. Moreover, activation of the S1P1 receptor promotes the migration of endothelial cells and lymphocytes, whereas the S1P2 receptor exerts an antagonistic effect. This receptor interplay plays a significant role in regulating the localization and migration of immune cells [135]. Additionally, Tiper and Baeyens et al. highlighted that the interaction between S1P and its receptors is critical for the egress of immune cells from lymphoid organs, serving as a key mechanism for maintaining the effective distribution of immune cells in peripheral tissues [136,137]. Pérez-Jeldres et al. further found that the gradient of S1P in tissues and blood, characterized by low levels in tissues and high levels in blood, plays an important regulatory role in lymphocyte migration. The enzymes responsible for the synthesis and degradation of S1P are essential for maintaining this gradient, thereby influencing the localization and migration of lymphocytes [124].

Secondly, S1P reduces the release of inflammatory factors by inhibiting inflammatory signaling pathways. For instance, S1P can inhibit the activation of NF-κB through the activation of the S1PR1 receptor. NF-κB is a critical transcription factor that regulates the expression of various inflammatory factors, including TNF-α and IL-6. Research indicates that S1P decreases the nuclear translocation of NF-κB, thereby lowering the production of these inflammatory mediators [124,135]. Additionally, S1P influences the MAPK signaling pathway, further inhibiting the synthesis of inflammation-related factors. Studies have shown that S1P reduces the release of pro-inflammatory cytokines by modulating ERK, JNK, and p38 MAPK signaling pathways [97,138]. Furthermore, S1P significantly impacts cytokine signaling pathways; by interacting with cytokine receptors, S1P can inhibit the synthesis of pro-inflammatory cytokines and subsequently reduce the overall inflammatory response [123]. Therefore, S1P regulates signaling pathways such as NF-κB and MAPK, decreases the production of pro-inflammatory factors like TNF-α and IL-6, alleviates radiation-induced inflammation in testicular tissue, and protects testicular cells from radiation damage [18,29].

Furthermore, S1P upregulates the expressions of anti-inflammatory factors, such as IL-10 and PG_E2_, which inhibit inflammatory responses and promote tissue repair. By modulating the inflammatory response in this manner, S1P helps prevent excessive inflammatory damage [8,18]. IL-10 is a crucial anti-inflammatory cytokine; its increased expression can suppress inflammatory responses and facilitate tissue healing. Research indicates that S1P can polarize macrophages toward the M2 phenotype, enhancing IL-10 release and promoting the secretion of anti-inflammatory factors by tumor-associated macrophages (TAMs) [139]. PG_E2_ is another key anti-inflammatory mediator, and macrophages stimulated by S1P are capable of secreting PG_E2_, which induces endothelial cell migration and enhances angiogenesis, playing a significant role in tumor progression [139]. Moreover, in chronic inflammation and intestinal diseases such as inflammatory bowel disease, selective S1P receptor modulators are believed to exert anti-inflammatory effects without inhibiting epithelial repair [114]. Additionally, the dynamic changes in S1P metabolism and signal transduction following muscle injury or lesions indicate its potential role in tissue repair and regeneration [140].

Finally, S1P protects the function of vascular endothelial cells by promoting their survival and reducing inflammation-induced increases in vascular permeability, thereby mitigating tissue damage related to inflammation [8,28,34]. Research conducted by Hsiao and Xiong et al. showed that S1P promotes the proliferation and migration of endothelial cells, enhancing the stability and integrity of blood vessels [141,142]. Similarly, Mahajan Thakur and Mohammed et al. reported that S1P plays a crucial role in maintaining endothelial cell barrier function by reducing permeability and protecting blood vessels from damage [102,143]. Furthermore, Zhang and Burg et al. found that S1P activates downstream signaling by binding to S1P receptors, which enhances the survival and function of endothelial cells [144,145]. Hsiao et al. also noted that S1P can preserve anticoagulant signals in endothelial cells, such as maintaining Syndecan-1 (SDC1), thereby reducing the risk of thrombosis [141]. Additionally, agonists of the S1P1 receptor have been investigated for their ability to enhance the endothelial barrier, limiting the escape of white blood cells from capillaries and reducing inflammatory damage [146].

### 4.4. Long-Term Protective Effect of S1P on Testicular Function

S1P not only protects testicular tissue from acute injury but also facilitates the recovery of damaged tissue by modulating cell proliferation and repair mechanisms, thereby providing long-term protective effects [8]. Otala et al. found that S1P exerts a partial protective effect against radiation-induced death of testicular germ cells, with inhibition rates ranging from 16% to 47%. This suggests S1P is critical for the recovery of damaged testicular tissue and warrants further investigation [147]. Additional research indicates that the protective effect of S1P is manifested through its inhibition of germ cell death caused by external insults, such as radiation, providing a significant foundation for the recovery of testicular tissue [135]. Moreover, S1P promotes cell regeneration in damaged testicular tissue and aids in restoring normal function by regulating the proliferation of satellite cells [118]. Furthermore, S1P regulates cell proliferation, migration, and survival by activating various signaling pathways. The interplay of these mechanisms offers multi-level support for the repair of damaged testes [148,149].

Additionally, S1P contributes to male reproductive health by preserving endocrine function and supporting sperm production [2,11,17]. Research by Clavijo and Hsiao indicates that S1P significantly impacts the male endocrine system, particularly with regard to regulating hormone secretion. It can influence the levels of reproductive hormones, such as testosterone, which are crucial for maintaining normal reproductive function [150]. Furthermore, S1P is vital to spermatogenesis as it promotes the proliferation and differentiation of spermatogonia, thereby ensuring both the quantity and quality of sperm [17]. S1P is also involved in the maturation process of sperm, enhancing sperm motility and fertilization capability. This action may link the regulation of intracellular calcium ion concentration and energy metabolism, which directly affects sperm motility [17]. Additionally, Wang et al. suggest that S1P deficiency may lead to impaired sperm production, ultimately affecting male fertility [17].

## 5. Clinical Application Prospects and Challenges

As a prospective clinical therapeutic agent, S1P offers broad application potential but also faces certain challenges [2,9,87,106]. It demonstrates a notable protective effect against radiation-induced testicular injury. Research indicates that S1P alleviates radiation-induced testicular damage through multiple mechanisms, including antioxidant, anti-apoptotic, and anti-inflammatory effects [4,16,18]. For instance, Shen et al. reported that S1P can mitigate radiation damage to the testes and intestines by regulating the expression of antioxidant enzymes, thereby exerting its protective effect against radiation [151]. Ran et al. emphasized the unique role of S1P in radiation damage, comparing its effects with those of other known antioxidants, such as selenium and metoprolol. Their results showed that S1P effectively activates the Nrf2 antioxidant signaling pathway while inhibiting NF-κB, which further reduces radiation damage to the testes [152]. Additionally, Liu et al. found that S1P and its analogs (such as FTY720/Fingolimod) can preserve testicular and ovarian function and fertility in both animals and humans exposed to radiation, highlighting S1P’s protective role against radiation-induced germ cell damage, particularly in reducing cell apoptosis [153]. Moreover, S1P can regulate key inflammatory signaling pathways, such as NF-κB, inhibit pro-inflammatory cytokines, and mitigate cell damage caused by inflammatory responses [147,154].

In vitro studies demonstrate that S1P significantly reduces radiation-induced ROS generation, inhibits cell apoptosis, and decreases the levels of inflammatory factors, thereby preserving the integrity and function of testicular cells [5,34]. Shen et al. investigated the protective effect of grape seed anthocyanins (GSPs) on radiation- induced testicular and intestinal damage and found that GSPs could significantly reduce ROS levels in TM3 and HIEC cells following radiation exposure [151]. Furthermore, another study suggests that abnormal changes in sperm induced by radiation may be associated with increased intracellular ROS, further supporting the potential role of S1P as an antioxidant capable of restoring sperm quality and function [155].

In animal studies, the therapeutic effects of S1P are notable, as it enhances sperm quality following radiation injury, improves testicular tissue structure, and reduces post-injury inflammatory responses [2,7,8]. Research has shown that radiation can significantly diminish sperm motility, concentration, and vitality while increasing the number of abnormal sperm. However, S1P has been found to alleviate these detrimental effects by protecting testicular structure, increasing sperm density, improving sperm motility, and reducing deformity rates [156,157,158]. Al Wattar et al. demonstrated that S1P offers unique advantages in combating oxidative stress and improving sperm quality. Compared to traditional antioxidants, S1P may be more effective in promoting testicular repair and facilitating sperm recovery [159]. Furthermore, Majzoub et al. suggested that S1P could provide a novel therapeutic option for treating radiation injuries, particularly with regard to protecting sperm and testicular tissue [160]. These findings provide compelling evidence of S1P’s ability to protect the testes from radiation damage and underscore its therapeutic potential.

To explore the potential clinical applications of S1P, further research is essential to assess its safety, optimize dosage, and evaluate its potential synergistic use with other treatment modalities [2,17,106]. S1P and its derivatives may emerge as novel therapies for treating radiation-induced testicular injury, offering new strategies for safeguarding male reproductive health in clinical settings. Notably, the role of S1P in cancer, atherosclerosis, diabetes, osteoporosis, and other pathological processes has been extensively studied, highlighting its central role in various diseases and providing important theoretical support for the development of S1P-based drugs [20,99]. Future research should focus on elucidating the specific mechanisms and therapeutic effects of S1P and its derivatives in the repair of testicular radiation injury. This research may encompass both animal experiments and clinical trials to thoroughly evaluate their safety and efficacy.

## 6. Conclusions

The potential clinical applications of S1P and its derivatives are garnering increasing attention, particularly regarding their role in enhancing male reproductive health. Research indicates that S1P has significant potential to protect testicular tissue and improve sperm quality through the regulation of various mechanisms, including cell signaling pathways, antioxidant activity, anti-apoptotic effects, and anti-inflammatory responses [10,54,56]. In clinical settings, S1P and its derivatives may offer a novel therapeutic strategy for preventing and treating testicular injury resulting from radiation, chemotherapy, or other factors [9,14,20]. Firstly, S1P and its analogs can effectively mitigate radiation-induced testicular damage by reducing oxidative stress, inhibiting cell apoptosis, and alleviating inflammatory responses, thereby preserving testicular function and supporting sperm production. Secondly, drug delivery systems based on S1P, such as nanocarriers or targeted delivery technologies, are anticipated to enhance bioavailability and targeting, thereby improving therapeutic efficacy. Future research should emphasize dose optimization, long-term safety assessment, and the combined use of S1P and its derivatives with other therapeutic approaches in clinical settings. Moreover, further exploration of the specific mechanisms underlying S1P’s action on male reproductive health will aid in the development of more effective treatments. In summary, S1P and its derivatives offer novel therapeutic strategies for enhancing male reproductive health and are anticipated to play a significant role in clinical practice.

## Data Availability

Not applicable.

## Abbreviations

Akt/PKB: protein kinase B, BTB: blood–testis barrier, CDK: cyclin-dependent kinase, DSB: DNA double-strand break, EGF: epidermal growth factor, ERK: extracellular signal-regulated kinase, GPx: glutathione peroxidase, H_2_O_2_: hydrogen peroxide, HIF-1α: hypoxia inducible factor-1 alpha, IL-6: interleukin-6, IL-10: interleukin-10, MAPK: mitogen-activated protein kinase, NF-κB: nuclear factor kappa B, NGF: nerve growth factor, PG_E2_: prostaglandin E2, PI-3K: phosphatidylinositol-3 kinase, ROS: reactive oxygen species, S1P: sphingosine 1-phosphate, SOD: superoxide dismutase, SphK: sphingosine kinase, SPL: S1P lyase, SSC: spermatogonial stem cell, O^2−^: superoxide anion, TAM: tumor-associated macrophage, TGF-β: transforming growth factor beta, TNF-α: tumor necrosis factor alpha, TRAF2: tumor necrosis factor receptor-associated factor 2.

## References

[1] Azmoonfar R., Moslehi M., Shahbazi-Gahrouei D. (2024). Radioprotective effect of selenium nanoparticles: A mini review. IET Nanobiotechnol..

[2] Spadella M.A., Silva E.J.R., Chies A.B., Almeida L.A. (2024). Insights into antioxidant strategies to counteract radiation-induced male infertility. Antioxid. Redox Signal..

[3] Kubikova E., Klein M., Svitok P., Stefanic J., Benus R., Polak S., Varga I. (2019). Fertility maintenance in male oncological patients: Current state and future perspectives. Bratisl. Lek. Listy.

[4] Skrzypek M., Wdowiak A., Panasiuk L., Stec M., Szczygieł K., Zybała M., Filip M. (2019). Effect of ionizing radiation on the female reproductive system. Ann. Agric. Environ. Med..

[5] Yang J., Ou X., Shu M., Wang J., Zhang X., Wu Z., Hao W., Zeng H., Shao L. (2024). Inhibition of p38MAPK signalling pathway alleviates radiation-induced testicular damage through improving spermatogenesis. Br. J. Pharmacol..

[6] Xu R., Shen S., Wang D., Ye J., Song S., Wang Z., Yue Z. (2023). The role of HIF-1α-mediated autophagy in ionizing radiation-induced testicular injury. J. Mol. Histol..

[7] Said R.S., Mohamed H.A., Kassem D.H. (2020). Alpha-lipoic acid effectively attenuates ionizing radiation-mediated testicular dysfunction in rats: Crosstalk of NF-ĸB, TGF-β, and PPAR-ϒ pathways. Toxicology.

[8] Shokri M., Shamsaei M.E., Malekshah A.K., Amiri F.T. (2020). The protective effect of melatonin on radiofrequency electromagnetic fields of mobile phone-induced testicular damage in an experimental mouse model. Andrologia.

[9] Amer M.E., Othman A.I., Abozaid H.M., El-Missiry M.A. (2022). Utility of melatonin in mitigating ionizing radiation-induced testis injury through synergistic interdependence of its biological properties. Biol. Res..

[10] Tajabadi E., Javadi A., Azar N.A., Najafi M., Shirazi A., Shabeeb D., Musa A.E. (2020). Radioprotective effect of a combination of melatonin and metformin on mice spermatogenesis: A histological study. Int. J. Reprod. Biomed..

[11] Take G., Erdogan D., Helvacioglu F., Göktas G., Ozbey G., Uluoglu C., Yücel B., Guney Y., Hicsonmez A., Ozkan S. (2009). Effect of melatonin and time of administration on irradiation-induced damage to rat testes. Braz. J. Med. Biol. Res..

[12] Xu R., Wang F., Zhang Z., Zhang Y., Tang Y., Bi J., Shi C., Wang D., Yang H., Wang Z. (2023). Diabetes-Induced Autophagy Dysregulation Engenders Testicular Impairment via Oxidative Stress. Oxid. Med. Cell. Longev..

[13] Rakici S.Y., Guzel A.I., Tumkaya L., Nalkiran H.S., Mercantepe T. (2020). Pelvic radiation-induced testicular damage: An experimental study at 1 gray. Syst. Biol. Reprod. Med..

[14] Fukunaga H., Butterworth K.T., Yokoya A., Ogawa T., Prise K.M. (2017). Low-dose radiation-induced risk in spermatogenesis. Int. J. Radiat. Biol..

[15] Gao X.F., Wang S.M., Peng R.Y., Gao Y.B., Li X., Dong H.Y., Ma J.J. (2008). High power microwave radiation damages blood-testis barrier in rats. Zhonghua Nan Ke Xue.

[16] Delic J.I., Hendry J.H., Morris I.D., Shalet S.M. (1986). Dose and time relationships in the endocrine response of the irradiated adult rat testis. J. Androl..

[17] Wang D., Tang Y., Wang Z. (2023). Role of sphingolipid metabolites in the homeostasis of steroid hormones and the maintenance of testicular functions. Front. Endocrinol. (Lausanne).

[18] Obinata H., Hla T. (2019). Sphingosine 1-phosphate and inflammation. Int. Immunol..

[19] Mendelson K., Evans T., Hla T. (2014). Sphingosine 1-phosphate signalling. Development.

[20] Cartier A., Hla T. (2019). Sphingosine 1-phosphate: Lipid signaling in pathology and therapy. Science.

[21] Tsai H.C., Han M.H. (2016). Sphingosine-1-phosphate (S1P) and S1P signaling pathway: Therapeutic targets in autoimmunity and inflammation. Drugs.

[22] Rutherford C., Childs S., Ohotski J., McGlynn L., Riddick M., MacFarlane S., Tasker D., Pyne S., Pyne N.J., Edwards J. (2013). Regulation of cell survival by sphingosine-1-phosphate receptor S1P1 via reciprocal ERK-dependent suppression of Bim and PI-3-kinase/protein kinase C-mediated upregulation of Mcl-1. Cell Death Dis..

[23] Usui S., Sugimoto N., Takuwa N., Sakagami S., Takata S., Kaneko S., Takuwa Y. (2004). Blood lipid mediator sphingosine 1-phosphate potently stimulates platelet-derived growth factor-A and -B chain expression through S1P1-Gi-Ras-MAPK-dependent induction of Kruppel-like factor 5. J. Biol. Chem..

[24] Pasternack D.A., Sharma A.I., Olson C.L., Epting C.L., Engman D.M. (2015). Sphingosine kinase regulates microtubule dynamics and organelle positioning necessary for proper G1/S cell cycle transition in trypanosoma brucei. mBio.

[25] Jarman K.E., Moretti P.A., Zebol J.R., Pitson S.M. (2010). Translocation of sphingosine kinase 1 to the plasma membrane is mediated by calcium- and integrin-binding protein 1. J. Biol. Chem..

[26] Czubowicz K., Strosznajder R. (2014). Ceramide in the molecular mechanisms of neuronal cell death. The role of sphingosine-1-phosphate. Mol. Neurobiol..

[27] Maceyka M., Payne S.G., Milstien S., Spiegel S. (2002). Sphingosine kinase, sphingosine-1-phosphate, and apoptosis. Biochim. Biophys. Acta.

[28] Pyne N.J., Buri A.E., Adams D.R., Pyne S. (2018). Sphingosine 1-phosphate and cancer. Adv. Biol. Regul..

[29] Ogretmen B. (2018). Sphingolipid metabolism in cancer signalling and therapy. Nat. Rev. Cancer.

[30] Pyne N.J., Pyne S. (2010). Sphingosine 1-phosphate and cancer. Nat. Rev. Cancer.

[31] Sordillo P.P., Sordillo D.C., Helson L. (2015). Review: The prolonged QT interval: Role of pro-inflammatory cytokines, reactive oxygen species and the ceramide and sphingosine-1 phosphate pathways. In Vivo.

[32] Chailapakul P., Kato T.A. (2022). From Basic Radiobiology to Translational Radiotherapy. Int. J. Mol. Sci..

[33] Hasaballah A.I. (2021). Impact of paternal transmission of gamma radiation on reproduction, oogenesis, and spermatogenesis of the housefly, *Musca domestica* L. (*Diptera: Muscidae*). Int. J. Radiat. Biol..

[34] Srivasatav S., Mishra J., Keshari P., Verma S., Aditi R. (2022). Impact of radiation on male fertility. Adv. Exp. Med. Biol..

[35] Asadi N., Bahmani M., Kheradmand A., Rafieian-Kopaei M. (2017). The Impact of Oxidative Stress on Testicular Function and the Role of Antioxidants in Improving it: A Review. J. Clin. Diagn. Res..

[36] Fukunaga H., Kaminaga K., Sato T., Butterworth K.T., Watanabe R., Usami N., Ogawa T., Yokoya A., Prise K.M. (2019). High-precision microbeam radiotherapy reveals testicular tissue-sparing effects for male fertility preservation. Sci. Rep..

[37] Qu N., Itoh M., Sakabe K. (2019). Effects of Chemotherapy and Radiotherapy on Spermatogenesis: The Role of Testicular Immunology. Int. J. Mol. Sci..

[38] De Felice F., Marchetti C., Marampon F., Cascialli G., Muzii L., Tombolini V. (2019). Radiation effects on male fertility. Andrology.

[39] Ibrahim A.A., Karam H.M., Shaaban E.A., Safar M.M., El-Yamany M.F. (2019). MitoQ ameliorates testicular damage induced by gamma irradiation in rats: Modulation of mitochondrial apoptosis and steroidogenesis. Life Sci..

[40] Hau R.K., Wright S.H., Cherrington N.J. (2023). In vitro and in vivo models for drug transport across the blood-testis barrier. Drug Metab. Dispos..

[41] Luaces J.P., Toro-Urrego N., Otero-Losada M., Capani F. (2023). What do we know about blood-testis barrier? current understanding of its structure and physiology. Front. Cell Dev. Biol..

[42] Wanjari U.R., Gopalakrishnan A.V. (2024). Blood-testis barrier: A review on regulators in maintaining cell junction integrity between Sertoli cells. Cell Tissue Res..

[43] Son Y., Heo K., Bae M.J., Lee C.G., Cho W.S., Kim S.D., Yang K., Shin I.S., Lee M.Y., Kim J.S. (2015). Injury to the blood-testis barrier after low-dose-rate chronic radiation exposure in mice. Radiat. Prot. Dosim..

[44] Zhang T., Liu T., Shao J., Sheng C., Hong Y., Ying W., Xia W. (2015). Antioxidant protects blood-testis barrier against synchrotron radiation X-ray-induced disruption. Spermatogenesis.

[45] Marjault H.B., Allemand I. (2016). Consequences of irradiation on adult spermatogenesis: Between infertility and hereditary risk. Mutat. Res. Rev. Mutat. Res..

[46] Dutta S., Sengupta P., Slama P., Roychoudhury S. (2021). Oxidative stress, testicular inflammatory pathways, and male reproduction. Int. J. Mol. Sci..

[47] Grewenig A., Schuler N., Rübe C.E. (2015). Persistent DNA damage in spermatogonial stem cells after fractionated low-dose irradiation of testicular tissue. Int. J. Radiat. Oncol. Biol. Phys..

[48] Cordelli E., Fresegna A.M., Leter G., Eleuteri P., Spanò M., Villani P. (2003). Evaluation of DNA damage in different stages of mouse spermatogenesis after testicular X irradiation. Radiat. Res..

[49] Guo C., Wang Q., Shuai P., Wang T., Wu W., Li Y., Huang S., Yu J., Yi L. (2024). Radiation and male reproductive system: Damage and protection. Chemosphere.

[50] Fukunaga H., Yokoya A., Prise K.M. (2022). A brief overview of radiation-induced effects on spermatogenesis and oncofertility. Cancers.

[51] Panganiban R.A., Snow A.L., Day R.M. (2013). Mechanisms of radiation toxicity in transformed and non-transformed cells. Int. J. Mol. Sci..

[52] Yin J., Ni B., Tian Z.Q., Yang F., Liao W.G., Gao Y.Q. (2017). Regulatory effects of autophagy on spermatogenesis. Biol. Reprod..

[53] La H.M., Hobbs R.M. (2019). Mechanisms regulating mammalian spermatogenesis and fertility recovery following germ cell depletion. Cell. Mol. Life Sci..

[54] Liu W., Du L., Li J., He Y., Tang M. (2024). Microenvironment of spermatogonial stem cells: A key factor in the regulation of spermatogenesis. Stem Cell Res. Ther..

[55] Schmitt D.A., Ullrich S.E. (2000). Exposure to ultraviolet radiation causes dendritic cells/macrophages to secrete immune-suppressive IL-12p40 homodimers. J. Immunol..

[56] Khan M.G.M., Wang Y. (2022). Advances in the current understanding of how low-dose radiation affects the cell cycle. Cells.

[57] Haines G.A., Hendry J.H., Daniel C.P., Morris I.D. (2002). Germ cell and dose-dependent DNA damage measured by the comet assay in murine spermatozoaa after testicular X-irradiation. Biol. Reprod..

[58] Kesari K.K., Agarwal A., Henkel R. (2018). Radiations and male fertility. Reprod. Biol. Endocrinol..

[59] Liu X., Chen Q., Ding X., Zhao Y., Zhang K., Yu P., Cui F., Xue B. (2019). X-ray-induced reproductive dysfunction and differentially expressed piRNAs in male mice. Hum. Exp. Toxicol..

[60] Li H.Y., Zhang H., Miao G.Y., Xie Y., Sun C., Di C.X., Liu Y., Liu Y.Y., Zhang X., Ma X.F. (2013). Simulated microgravity conditions and carbon ion irradiation induce spermatogenic cell apoptosis and sperm DNA damage. Biomed. Environ. Sci..

[61] Yang J., Xu R., Luan Y., Fan H., Yang S., Liu J., Zeng H., Shao L. (2022). Rapamycin Ameliorates Radiation-Induced Testis Damage in Mice. Front. Cell Dev. Biol..

[62] Baliga S., Patel S., Naqa I.E., Li X.A., Cohen L.E., Howell R.M., Hoppe B.S., Constine L.S., Palmer J.D., Hamstra D. (2024). Testicular dysfunction in male childhood cancer survivors treated with radiation therapy: A PENTEC comprehensive review. Int. J. Radiat. Oncol. Biol. Phys..

[63] Peak T.C., Haney N.M., Wang W., DeLay K.J., Hellstrom W.J. (2016). Stem cell therapy for the treatment of Leydig cell dysfunction in primary hypogonadism. World J. Stem Cells.

[64] Delic J.I., Hendry J.H., Morris I.D., Shalet S.M. (1986). Leydig cell function in the pubertal rat following local testicular irradiation. Radiother. Oncol..

[65] Georgakopoulos I., Kouloulias V., Ntoumas G.N., Desse D., Koukourakis I., Kougioumtzopoulou A., Kanakis G., Zygogianni A. (2024). Radiotherapy and testicular function: A comprehensive review of the radiation-induced effects with an emphasis on spermatogenesis. Biomedicines.

[66] Sivakumar R., Sivaraman P.B., Mohan-Babu N., Jainul-Abideen I.M., Kalliyappan P., Balasubramanian K. (2006). Radiation exposure impairs luteinizing hormone signal transduction and steroidogenesis in cultured human leydig cells. Toxicol. Sci..

[67] Yamasaki H., Sandrof M.A., Boekelheide K. (2010). Suppression of radiation-induced testicular germ cell apoptosis by 2,5-hexanedione pretreatment. I. Histopathological analysis reveals stage dependence of attenuated apoptosis. Toxicol. Sci..

[68] Hedger M.P. (2011). Immunophysiology and pathology of inflammation in the testis and epididymis. J. Androl..

[69] Gong J., Zeng Q., Yu D., Duan Y.G. (2020). T Lymphocytes and testicular immunity: A new insight into immune regulation in testes. Int. J. Mol. Sci..

[70] Bhushan S., Theas M.S., Guazzone V.A., Jacobo P., Wang M., Fijak M., Meinhardt A., Lustig L. (2020). Immune cell subtypes and their function in the testis. Front. Immunol..

[71] Zhao S., Zhu W., Xue S., Han D. (2014). Testicular defense systems: Immune privilege and innate immunity. Cell. Mol. Immunol..

[72] Zhu Q., Xia M., Wang Z., Li P.L., Li N. (2011). A novel lipid natriuretic factor in the renal medulla: Sphingosine-1-phosphate. Am. J. Physiol. Renal Physiol..

[73] Zhang X., Ritter J.K., Li N. (2018). Sphingosine-1-phosphate pathway in renal fibrosis. Am. J. Physiol. Renal Physiol..

[74] Cannavo A., Liccardo D., Komici K., Corbi G., de Lucia C., Femminella G.D., Elia A., Bencivenga L., Ferrara N., Koch W.J. (2017). Sphingosine kinases and sphingosine 1-phosphate receptors: Signaling and actions in the cardiovascular system. Front. Pharmacol..

[75] Alkafaas S.S., Elsalahaty M.I., Ismail D.F., Radwan M.A., Elkafas S.S., Loutfy S.A., Elshazli R.M., Baazaoui N., Ahmed A.E., Hafez W. (2024). The emerging roles of sphingosine 1-phosphate and SphK1 in cancer resistance: A promising therapeutic target. Cancer Cell Int..

[76] Li Q., Qian J., Li Y., Huang P., Liang H., Sun H., Liu C., Peng J., Lin X., Chen X. (2020). Generation of sphingosine-1-phosphate by sphingosine kinase 1 protects nonalcoholic fatty liver from ischemia/reperfusion injury through alleviating reactive oxygen species production in hepatocytes. Free Radic. Biol. Med..

[77] Sukocheva O.A., Neganova M.E., Aleksandrova Y., Burcher J.T., Chugunova E., Fan R., Tse E., Sethi G., Bishayee A., Liu J. (2024). Signaling controversy and future therapeutical perspectives of targeting sphingolipid network in cancer immune editing and resistance to tumor necrosis factor-α immunotherapy. Cell Commun. Signal..

[78] Kajita K., Ishii I., Mori I., Asano M., Fuwa M., Morita H. (2024). Sphingosine 1-phosphate regulates obesity and glucose homeostasis. Int. J. Mol. Sci..

[79] Lebman D.A., Spiegel S. (2008). Cross-talk at the crossroads of sphingosine-1-phosphate, growth factors, and cytokine signaling. J. Lipid Res..

[80] Bu Y., Wu H., Deng R., Wang Y. (2021). Therapeutic Potential of SphK1 Inhibitors Based on Abnormal Expression of SphK1 in Inflammatory Immune Related-Diseases. Front. Pharmacol..

[81] Lowe N.G., Swaney J.S., Moreno K.M., Sabbadini R.A. (2009). Sphingosine-1-phosphate and sphingosine kinase are critical for transforming growth factor-beta-stimulated collagen production by cardiac fibroblasts. Cardiovasc. Res..

[82] Spiegel S., Milstien S. (2011). The outs and the ins of sphingosine-1-phosphate in immunity. Nat. Rev. Immunol..

[83] Chen H., Haddadi N., Zhu X., Hatoum D., Chen S., Nassif N.T., Lin Y., McGowan E.M. (2022). Expression profile of sphingosine kinase 1 isoforms in human cancer tissues and cells: Importance and clinical relevance of the neglected 1b-isoform. J. Oncol..

[84] Alemany R., van Koppen C.J., Danneberg K., Braak M.T., Heringdorf D.M.Z. (2007). Regulation and functional roles of sphingosine kinases. Naunyn Schmiedebergs Arch. Pharmacol..

[85] Nayak D., Huo Y., Kwang W.X., Pushparaj P.N., Kumar S.D., Ling E.A., Dheen S.T. (2010). Sphingosine kinase 1 regulates the expression of proinflammatory cytokines and nitric oxide in activated microglia. Neuroscience.

[86] Kim S., Sieburth D. (2018). Sphingosine kinase regulates neuropeptide secretion during the oxidative stress-response through intertissue signaling. J. Neurosci..

[87] Hu Y., Dai K. (2022). Sphingosine 1-phosphate metabolism and signaling. Adv. Exp. Med. Biol..

[88] Ren R., Pang B., Han Y., Li Y. (2021). A Glimpse of the structural biology of the metabolism of sphingosine-1-phosphate. Contact.

[89] Leong W.I., Saba J.D. (2010). S1P metabolism in cancer and other pathological conditions. Biochimie.

[90] Saba J.D., Hla T. (2004). Point-counterpoint of sphingosine 1-phosphate metabolism. Circ. Res..

[91] George N., Xiao J. (2024). Inhibiting sphingosine 1-phosphate lyase: From efficacy to mechanism. Neurobiol. Dis..

[92] Olivera A., Allende M.L., Proia R.L. (2013). Shaping the landscape: Metabolic regulation of S1P gradients. Biochim. Biophys. Acta.

[93] Stepanovska B., Lange A.I., Schwalm S., Pfeilschifter J., Coldewey S.M., Huwiler A. (2020). Downregulation of S1P lyase improves barrier function in human cerebral microvascular endothelial cells following an inflammatory challenge. Int. J. Mol. Sci..

[94] Kumar A., Zamora-Pineda J., Degagné E., Saba J.D. (2017). S1P lyase regulation of thymic egress and oncogenic inflammatory signaling. Mediators Inflamm..

[95] Uranbileg B., Kurano M., Kano K., Sakai E., Arita J., Hasegawa K., Nishikawa T., Ishihara S., Yamashita H., Seto Y. (2022). Sphingosine 1-phosphate lyase facilitates cancer progression through converting sphingolipids to glycerophospholipids. Clin. Transl. Med..

[96] Maceyka M., Spiegel S. (2014). Sphingolipid metabolites in inflammatory disease. Nature.

[97] Sun G., Wang B., Wu X., Cheng J., Ye J., Wang C., Zhu H., Liu X. (2024). How do sphingosine-1-phosphate affect immune cells to resolve inflammation?. Front. Immunol..

[98] Gurgul-Convey E. (2022). To be or not to be: The divergent action and metabolism of sphingosine-1 phosphate in pancreatic beta-cells in response to cytokines and fatty acids. Int. J. Mol. Sci..

[99] Maceyka M., Harikumar K.B., Milstien S., Spiegel S. (2012). Sphingosine-1-phosphate signaling and its role in disease. Trends Cell Biol..

[100] Tolksdorf C., Moritz E., Wolf R., Meyer U., Marx S., Bien-Möller S., Garscha U., Jedlitschky G., Rauch B.H. (2022). Platelet-derived S1P and its relevance for the communication with immune cells in multiple human diseases. Int. J. Mol. Sci..

[101] Grassi S., Mauri L., Prioni S., Cabitta L., Sonnino S., Prinetti A., Giussani P. (2019). Sphingosine 1-phosphate receptors and metabolic enzymes as druggable targets for brain diseases. Front. Pharmacol..

[102] Mahajan-Thakur S., Böhm A., Jedlitschky G., Schrör K., Rauch B.H. (2015). Sphingosine-1-phosphate and its receptors: A mutual link between blood coagulation and inflammation. Mediat. Inflamm..

[103] Tabasinezhad M., Samadi N., Ghanbari P., Mohseni M., Saei A.A., Sharifi S., Saeedi N., Pourhassan A. (2013). Sphingosin 1-phosphate contributes in tumor progression. J. Cancer Res. Ther..

[104] Duan M., Gao P., Chen S.X., Novák P., Yin K., Zhu X. (2022). Sphingosine-1-phosphate in mitochondrial function and metabolic diseases. Obes. Rev..

[105] Chen W., Lu H., Yang J., Xiang H., Peng H. (2016). Sphingosine 1-phosphate in metabolic syndrome (Review). Int. J. Mol. Med..

[106] Wang X., Sun Y., Peng X., Naqvi S.M.A.S., Yang Y., Zhang J., Chen M., Chen Y., Chen H., Yan H. (2020). The tumorigenic effect of sphingosine kinase 1 and its potential therapeutic target. Cancer Control.

[107] Fan X., Liu L., Shi Y., Guo F., He X., Zhao X., Zhong D., Li G. (2021). Recent advances of the function of sphingosine 1-phosphate (S1P) receptor S1P3. J. Cell Physiol..

[108] Alaamery M., Albesher N., Aljawini N., Alsuwailm M., Massadeh S., Wheeler M.A., Chao C.C., Quintana F.J. (2021). Role of sphingolipid metabolism in neurodegeneration. J. Neurochem..

[109] Wieczorek I., Strosznajder R.P. (2023). Recent Insight into the Role of Sphingosine-1-Phosphate Lyase in Neurodegeneration. Int. J. Mol. Sci..

[110] Jozefczuk E., Guzik T.J., Siedlinski M. (2020). Significance of sphingosine-1-phosphate in cardiovascular physiology and pathology. Pharmacol. Res..

[111] Ouyang J., Shu Z., Chen S., Xiang H., Lu H. (2020). The role of sphingosine 1-phosphate and its receptors in cardiovascular diseases. J. Cell. Mol. Med..

[112] Castillo S.S., Teegarden D. (2003). Sphingosine-1-phosphate inhibition of apoptosis requires mitogen-activated protein kinase phosphatase-1 in mouse fibroblast C3H10T 1/2 cells. J. Nutr..

[113] Giussani P., Tringali C., Riboni L., Viani P., Venerando B. (2014). Sphingolipids: Key regulators of apoptosis and pivotal players in cancer drug resistance. Int. J. Mol. Sci..

[114] Zou F., Wang S., Xu M., Wu Z., Deng F. (2023). The role of sphingosine-1-phosphate in the gut mucosal microenvironment and inflammatory bowel diseases. Front. Physiol..

[115] Zhao X., Yang L., Chang N., Hou L., Zhou X., Yang L., Li L. (2020). Neutrophils undergo switch of apoptosis to NETosis during murine fatty liver injury via S1P receptor 2 signaling. Cell Death Dis..

[116] Nix M., Stoffel W. (2000). Perturbation of membrane microdomains reduces mitogenic signaling and increases susceptibility to apoptosis after T cell receptor stimulation. Cell Death Differ..

[117] Patwardhan G.A., Beverly L.J., Siskind L.J. (2016). Sphingolipids and mitochondrial apoptosis. J. Bioenerg. Biomembr..

[118] Zhang L., Dong Y., Wang Y., Hu W., Dong S., Chen Y. (2020). Sphingosine-1-phosphate (S1P) receptors: Promising drug targets for treating bone-related diseases. J. Cell Mol. Med..

[119] Xiao S., Peng K., Li C., Long Y., Yu Q. (2023). The role of sphingosine-1-phosphate in autophagy and related disorders. Cell Death Discov..

[120] Hait N.C., Oskeritzian C.A., Paugh S.W., Milstien S., Spiegel S. (2006). Sphingosine kinases, sphingosine 1-phosphate, apoptosis and diseases. Biochim. Biophys. Acta.

[121] Pan W., Hu L., Chen Y., Zhu Z., Wang Y., Song J., Shan Z. (2020). Sphingosine-1-phosphate alleviates irradiation-induced parotid injury in a miniature pig model. Oral Dis..

[122] Li S., Chen J., Fang X., Xia X. (2017). Sphingosine-1-phosphate activates the AKT pathway to inhibit chemotherapy induced human granulosa cell apoptosis. Gynecol. Endocrinol..

[123] Lucaciu A., Brunkhorst R., Pfeilschifter J.M., Pfeilschifter W., Subburayalu J. (2020). The S1P-S1PR axis in neurological disorders-Insights into current and future therapeutic perspectives. Cells.

[124] Pérez-Jeldres T., Alvarez-Lobos M., Rivera-Nieves J. (2021). Targeting sphingosine-1-phosphate signaling in immune-mediated diseases: Beyond multiple sclerosis. Drugs.

[125] Fei X., Huang J., Li F., Wang Y., Shao Z., Dong L., Wu Y., Li B., Zhang X., Lv B. (2023). The Scap-SREBP1-S1P/S2P lipogenesis signal orchestrates the homeostasis and spatiotemporal activation of NF-κB. Cell Rep..

[126] Wollny T., Wątek M., Durnaś B., Niemirowicz K., Piktel E., Żendzian-Piotrowska M., Góźdź S., Bucki R. (2017). Sphingosine-1-phosphate metabolism and its role in the development of inflammatory bowel disease. Int. J. Mol. Sci..

[127] Yu L., He L., Gan B., Ti R., Xiao Q., Hu H., Zhu L., Wang S., Ren R. (2022). Structural insights into sphingosine-1-phosphate receptor activation. Proc. Natl. Acad. Sci. USA.

[128] Sun K., Zhang Y., D’Alessandro A., Nemkov T., Song A., Wu H., Liu H., Adebiyi M., Huang A., Wen Y.E. (2016). Sphingosine-1-phosphate promotes erythrocyte glycolysis and oxygen release for adaptation to high-altitude hypoxia. Nat. Commun..

[129] Catarzi S., Romagnoli C., Marcucci G., Favilli F., Iantomasi T., Vincenzini M.T. (2011). Redox regulation of ERK1/2 activation induced by sphingosine 1-phosphate in fibroblasts: Involvement of NADPH oxidase and platelet-derived growth factor receptor. Biochim. Biophys. Acta.

[130] Gusti A.M.T., Qusti S.Y., Alshammari E.M., Toraih E.A., Fawzy M.S. (2021). Antioxidants-related superoxide dismutase (SOD), catalase (CAT), glutathione Peroxidase (GPX), glutathione-S-Transferase (GST), and nitric oxide synthase (NOS) gene variants analysis in an obese population: A preliminary case-control study. Antioxidants.

[131] Aitken R.J., Roman S.D. (2008). Antioxidant systems and oxidative stress in the testes. Oxid. Med. Cell. Longev..

[132] Arena S., Iacona R., Antonuccio P., Russo T., Salvo V., Gitto E., Impellizzeri P., Romeo C. (2017). Medical perspective in testicular ischemia-reperfusion injury. Exp. Ther. Med..

[133] Morris G., Gevezova M., Sarafian V., Maes M. (2022). Redox regulation of the immune response. Cell. Mol. Immunol..

[134] Bravo G.Á., Cedeño R.R., Casadevall M.P., Ramió-Torrentà L. (2022). Sphingosine-1-phosphate (S1P) and S1P signaling pathway modulators, from current insights to future perspectives. Cells.

[135] Chatzikonstantinou S., Poulidou V., Arnaoutoglou M., Kazis D., Heliopoulos I., Grigoriadis N., Boziki M. (2021). Signaling through the S1P-S1PR axis in the gut, the immune and the central nervous system in multiple sclerosis: Implication for pathogenesis and treatment. Cells.

[136] Tiper I.V., East J.E., Subrahmanyam P.B., Webb T.J. (2016). Sphingosine 1-phosphate signaling impacts lymphocyte migration, inflammation and infection. Pathog. Dis..

[137] Baeyens A.A.L., Schwab S.R. (2020). Finding a way out: S1P signaling and immune cell migration. Annu. Rev. Immunol..

[138] Sukocheva O.A., Furuya H., Ng M.L., Friedemann M., Menschikowski M., Tarasov V.V., Chubarev V.N., Klochkov S.G., Neganova M.E., Mangoni A.A. (2020). Sphingosine kinase and sphingosine-1-phosphate receptor signaling pathway in inflammatory gastrointestinal disease and cancers: A novel therapeutic target. Pharmacol. Ther..

[139] Rodriguez Y.I., Campos L.E., Castro M.G., Aladhami A., Oskeritzian C.A., Alvarez S.E. (2016). Sphingosine-1 phosphate: A new modulator of immune plasticity in the tumor microenvironment. Front. Oncol..

[140] Sassoli C., Pierucci F., Zecchi-Orlandini S., Meacci E. (2019). Sphingosine 1-phosphate (S1P)/ S1P receptor signaling and mechanotransduction: Implications for intrinsic tissue repair/regeneration. Int. J. Mol. Sci..

[141] Hsia K., Yang M.J., Chen W.M., Yao C.L., Lin C.H., Loong C.C., Huang Y.L., Lin Y.T., Lander A.D., Lee H. (2017). Sphingosine-1-phosphate improves endothelialization with reduction of thrombosis in recellularized human umbilical vein graft by inhibiting syndecan-1 shedding in vitro. Acta Biomater..

[142] Xiong Y., Hla T. (2014). S1P control of endothelial integrity. Curr. Top Microbiol. Immunol..

[143] Mohammed S., Bindu A., Viswanathan A., Harikumar K.B. (2023). Sphingosine 1-phosphate signaling during infection and immunity. Prog. Lipid Res..

[144] Zhang G., Yang L., Kim G.S., Ryan K., Lu S., O’Donnell R.K., Spokes K., Shapiro N., Aird W.C., Kluk M.J. (2013). Critical role of sphingosine-1-phosphate receptor 2 (S1PR2) in acute vascular inflammation. Blood.

[145] Burg N., Salmon J.E., Hla T. (2022). Sphingosine 1-phosphate receptor-targeted therapeutics in rheumatic diseases. Nat. Rev. Rheumatol..

[146] Burg N., Swendeman S., Worgall S., Hla T., Salmon J.E. (2018). Sphingosine 1-phosphate receptor 1 signaling maintains endothelial cell barrier function and protects against immune complex-induced vascular injury. Arthritis Rheumatol..

[147] Otala M., Suomalainen L., Pentikäinen M.O., Kovanen P., Tenhunen M., Erkkilä K., Toppari J., Dunkel L. (2004). Protection from radiation-induced male germ cell loss by sphingosine-1-phosphate. Biol. Reprod..

[148] Romani R., Manni G., Donati C., Pirisinu I., Bernacchioni C., Gargaro M., Pirro M., Calvitti M., Bagaglia F., Sahebkar A. (2018). S1P promotes migration, differentiation and immune regulatory activity in amniotic-fluid-derived stem cells. Eur. J. Pharmacol..

[149] Rivera J., Proia R.L., Olivera A. (2008). The alliance of sphingosine-1-phosphate and its receptors in immunity. Nat. Rev. Immunol..

[150] Clavijo R.I., Hsiao W. (2018). Update on male reproductive endocrinology. Transl. Androl. Urol..

[151] Shen H., Han J., Liu C., Cao F., Huang Y. (2022). Grape seed proanthocyanidins exert a radioprotective effect on the testes and intestines through antioxidant effects and inhibition of MAPK signal pathways. Front. Med. (Lausanne).

[152] Ran Y., Duan N., Gao Z., Liu Y., Liu X., Xue B. (2023). Sulforaphane attenuates irradiation induced testis injury in mice. Redox Rep..

[153] Liu L., Liang Z., Ma S., Li L., Liu X. (2023). Radioprotective countermeasures for radiation injury (Review). Mol. Med. Rep..

[154] Fettel J., Kühn B., Guillen N.A., Sürün D., Peters M., Bauer R., Angioni C., Geisslinger G., Schnütgen F., Heringdorf D.M.Z. (2019). Sphingosine-1-phosphate (S1P) induces potent anti-inflammatory effects in vitro and in vivo by S1P receptor 4-mediated suppression of 5-lipoxygenase activity. FASEB J..

[155] da Silva Mansano N., Jorge I.F., Chies A.B., Viani G.A., Spadella M.A. (2018). Effects of telmisartan and losartan on irradiated testes. Life Sci..

[156] Porubska B., Plevakova M., Fikarova N., Vasek D., Somova V., Sanovec O., Simonik O., Komrskova K., Krylov V., Tlapakova T. (2024). Therapeutic potential of Sertoli cells in vivo: Alleviation of acute inflammation and improvement of sperm quality. Stem Cell Res. Ther..

[157] Singh R.P., Escobar E., Wildt D., Patel S., Costa G.M.J., Pukazhenthi B. (2019). Effect of sphingosine-1-phosphate on cryopreserved sheep testicular explants cultured in vitro. Theriogenology.

[158] Ding J., Wang H., Wu Z.B., Zhao J., Zhang S., Li W. (2015). Protection of murine spermatogenesis against ionizing radiation-induced testicular injury by a green tea polyphenol. Biol. Reprod..

[159] Al Wattar B.H., Rimmer M.P., Teh J.J., Mackenzie S.C., Ammar O.F., Croucher C., Anastasiadis E., Gordon P., Pacey A., McEleny K. (2024). Pharmacological non-hormonal treatment options for male infertility: A systematic review and network meta-analysis. BMC Urol..

[160] Majzoub A., Agarwal A. (2018). Systematic review of antioxidant types and doses in male infertility: Benefits on semen parameters, advanced sperm function, assisted reproduction and live-birth rate. Arab. J. Urol..

